# Poor Lung Function Has Inverse Relationship with Microalbuminuria, an Early Surrogate Marker of Kidney Damage and Atherosclerosis: The 5^th^ Korea National Health and Nutrition Examination Survey

**DOI:** 10.1371/journal.pone.0094125

**Published:** 2014-04-09

**Authors:** Jin-Ha Yoon, Jong-Uk Won, Yeon-Soon Ahn, Jaehoon Roh

**Affiliations:** 1 The Institute for Occupational Health, Yonsei University College of Medicine, Seoul, Korea; 2 Department of Preventive Medicine and Public Health, Yonsei University College of Medicine, Seoul, Korea; 3 Graduate School of Public Health, Yonsei University College of Medicine, Seoul, Korea; 4 Department of Occupational and Environmental Medicine, Dongguk University Ilsan Hospital, Goyang, Korea; CUNY, United States of America

## Abstract

**Background:**

Despite epidemiological evidences of relationship between poor lung function and atherosclerosis, the relationship between poor lung function and microalbuminuria (MAU), an early surrogate marker of both kidney damage and atherosclerosis, is not well understood. Hence, we plan to investigate the relationship between poor lung function and MAU using multivariate models to adjust for other atherogenic risk factors.

**Methods:**

We used data from the 5^th^ Korean National Health and Nutrition Examination Survey. Poor lung function is determined by spirometric measurement, primarily through estimation of the forced vital capacity (FVC) and forced expiratory volume in 1 second (FEV1). Declines in the percent predicted FVC (<80%) and in the FEV_1_/FVC ratio (<0.7) are defined as restrictive and obstructive patterns, respectively. Urine albumin to urine creatinine levels ratio (UACR) were measured in spot urine samples. MAU was defined as UACR >30 mg/g.

**Results:**

Inverse relationship was observed between lung function and UACR. In an age-adjusted regression model, the regression coefficient (B) of 10% lower FVC was 11.09 in men (*P* = 0.002), which remained significant after adjustment for SBP, FBG, triglyceride level, BMI, smoking history, and heavy alcohol consumption (B = 7.52, *P* = 0.043). When the restrictive pattern was compared to the normal pattern, the odds ratios (OR) (95% confidence interval, 95%CI) for MAU were 1.90 (1.32–2.72) in men, after adjustment for age, hypertension, diabetes mellitus, triglyceride level, obesity, smoking history, physical activity, and heavy alcohol consumption.

**Conclusions:**

Our study, the first investigation in Asia, demonstrated that the restrictive pattern is related to MAU in men. Furthermore, there was linear relationship between lower FVC and UACR. Thus, our current study suggests that poor lung function, particularly the restrictive pattern, is related to kidney damage as well as atherosclerosis.

## Introduction

Microalbuminuria (MAU), as indicated by the urine albumin-to-creatinine ratio (UACR), occurs when blood albumin leaks from the kidneys into the urine [1]. This leakage also represents underlying problems in blood vessels and specifically endothelial dysfunction, a key mechanism of atherosclerosis [2]. Therefore, microalbuminuria functions as an early surrogate marker of kidney damage as well as a strong risk predictor of atherosclerosis or cardiovascular diseases [3]. Furthermore, the strong predictive power of MAU for the risk of atherosclerosis is independent of conventional cardiovascular risk factors, even in diabetes mellitus and hypertension patients [4].

Multiple pathological steps such as retention, modification, and accumulation of lipids in the endothelium occur prior to vascular damage or atherosclerosis, all of which are negatively influenced by systemic inflammation [5]. Systemic inflammation is present in various chronic disease states, including poor lung function [6]. Strong evidence indicates that poor lung function is a predictor of the morbidity and mortality of atherogenic events [7], and systemic inflammatory mechanisms are regarded as key mediators of the strong relationship between poor lung function and atherosclerosis [8].

Poor lung function is determined by spirometric measurement of dynamic lung capacity, primarily through estimation of the forced vital capacity (FVC) and forced expiratory volume in 1 second (FEV1). Clinically, a decline in the percent predicted FVC (<80%) and in the FEV_1_/FVC ratio (<0.7) are key phenotypes of restrictive and obstructive patterns of poor lung function, respectively. Both poor lung function patterns worsen detoxification of systemic inflammation brought on by physical activity, eventually resulting in persistent inflammatory status even after rest [9]. Furthermore, when the inflammatory status persists in the lung, a “spilling over” of inflammatory mediators from the lung to systemic circulation occurs [10]. Eventually, extrapulmonary organs such as bone, brain, and peripheral vasculature are affected by inflammatory mediators arising from the lungs [11]. In epidemiology studies, reduced FEV_1_ [12] as well as reduced FVC [13] were linked to atherogenic mortality. Chronic obstructive pulmonary disease (COPD), diagnosed when FEV1/FVC ratio is <0.7, is also a manifestation of poor lung function status well known to affect mortality due to atherogenic events [14].

Despite the epidemiological evidence of a relationship between poor lung function and the risk of atherosclerosis, the relationship between poor lung function and MAU, as an early surrogate marker of both kidney damage and atherosclerosis, has not been investigated. Tobacco smoking is a strong risk factor of both MAU and poor lung function [15], and obesity, hypertension, and diabetes mellitus are also risk factors of MAU. Therefore, whether poor lung function is related to MAU after controlling for smoking history, obesity, hypertension, and diabetes mellitus in multivariate models remains unknown.

The aim of our study was to investigate the relationship between poor lung function and MAU as an early surrogate marker of kidney damage as well as atherosclerosis and thus, to generate important information concerning the association between poor lung function and MAU in an Asian population. We used a multivariate model to adjust for obesity, hypertension, and diabetes mellitus as well as the lifestyle factors of smoking history, heavy alcohol consumption, and regular physical activity.

## Methods

### Ethics Statement

We used data from the 5^th^ Korean National Health and Nutrition Examination Survey (KNHANES). Data from 2011 to 2012 were selected for analysis. All voluntarily engaged participants provided written informed consent prior to their enrollment. The participants’ records, except survey date and home region, were anonymized prior to analysis. This study was approved by the Institutional Review Board (IRB) of Korea Centers for Disease Control and Prevention (IRB: 2011-02CON-06-C, 2012-01EXP-01-2C).

### 5th KNHANE from 2010 to 2012

The Korea Centers for Disease Control and Prevention conducted the 5^th^ KNHANES from 2010 to 2012. A geographic region based-multistage probability sampling of household units, stratified according to sex and age, was undertaken throughout the entire Korea population. Using the above sampling method, 192 household units consisting of 3,800 individuals were selected each year, resulting in a total of 576 units with 11,400 individuals included in the 5^th^ KNHANES from 2010 to 2012. The response rates in 2010, 2011, and 2012 were 81.9%, 80.4%, and 80.0%, respectively. The KNHANES comprises 3 major examinations. The first is a clinical health examination, including blood and urine biochemistry analyses, blood pressure assessment, pulmonary function tests, and roentgenography. The second is a comprehensive self-reported questionnaire of anthropometric and demographic characteristics such as health-related behaviors. The third is a questionnaire on 24-hour recall of nutritional information.

### Pulmonary Function Testing and Poor Lung Function Patterns

Spirometry tests to determine lung function patterns were performed by trained technician using dry rolling-seal spirometers (SensorMedics, Yorba Linda, USA) in participants older than 40 y [16]. The technicians were trained according to American Thoracic Society/European Respiratory Society criteria to review the spirometry test results. Two criteria were applied to spirometry data: (1) two or more acceptable spirometry curves to ensure correct inspiration and 6-s expiration and (2) <150-ml inter-measurement variability in FVC and FEV1. The spirometry tests were undertaken without a bronchodilator. Age, sex and height adjusted *normal predicted values of FVC and FEV1 in Korean general population were used to calculate the values of % predicted FVC and FEV_1_* [17]. The restrictive pattern was defined as percent predicted value of FVC <80%, and the obstructive airflow pattern was defined as FEV_1_/FVC ratio <0.7.

### Smoking History, Physical Activity, Heavy Alcohol Consumption, and Obesity

Smoking status was categorized as non-smoker, former smoker, and current smoker using a self-reported questionnaire. “Non-smoker” was defined as a lifetime history of smoking fewer than 100 cigarettes. Regular physical activity was defined as more than 20 min of physical activity sufficient to cause sweating, 3 or more times per week. Heavy alcohol consumption was defined as drinking 7 or more glasses of alcohol on 2 or more occasions per a week in men, and 5 or more glasses on 2 or more occasions per a week in women.

### Hypertension, Diabetes Mellitus, and Obesity

Hypertension was defined as blood pressure controlled using pharmacological treatment or systolic blood pressure (SBP) or diastolic blood pressure (DBP) exceeding 140 mmHg or 90 mmHg, respectively. Normal blood pressure was defined as SBP below 120 mmHg and DBP below 80 mmHg. Participants who fell below the hypertension threshold but above the threshold for normal blood pressure were defined as prehypertension. Diabetes mellitus was determined based on 8-h fasting blood glucose (FBG). FBG levels less than 100 mg/dl, 100–125 mg/dl, and 126 or above were defined as normal, glucose intolerance, and diabetes mellitus, respectively. Participant with diabetes mellitus controlled by pharmacological treatment were categorized as diabetes mellitus regardless of FBG level. Body mass index (BMI), the ratio of weight to squared height, was used for obesity parameters [18], with underweight (<18.5 kg/m^2^), normal (18.5–25.0 kg/m^2^), and overweight (≥25.0 kg/m^2^) categorized as dummy variables.

### Urine Analysis for MAU

Spot urine samples were used to analyze MAU in KNHANES [19]. Urine albumin and urine creatinine levels were measured in spot urine samples, from which UACR was estimated. Urine albumin and creatinine were determined using turbidimetric and kinetic colorimetric assays, respectively, using a Hitachi Automatic Analyzer 7600 (Hitachi, Japan). MAU was defined as UACR >30 mg albumin/g creatinine.

### Study Population for Data Analyses

Because UACR was measured from 2011 to 2012, 6,053 participants older than 40 y who completed UACR measurement and spirometry testing were selected for analysis (2,885 in 2011 and 3,168 in 2012). We excluded 33 asthma patients receiving pharmacological treatment, because spirometry was not undertaken after bronchodilation. Ultimately, a total of 6,020 participants (2,643 male and 3,377 female) were included in data analyses.

### Statistical Analysis

Frequency with percentage, mean with standard deviation (SD), or median with interquartile range was used to interpret the participant characteristics according to non-MAU vs. MAU status as appropriate. Furthermore, age adjusted comparisons were undertaken by using generalized linear model in table 1. Regression coefficients of UACR were calculated with respect to 10% lower percent predicted FVC and FEV_1_ values in multiple regression models. Odds ratios (ORs) with 95% confidence intervals (95%CI) were calculated in multiple logistic regression models according to poor lung function pattern (normal vs. restrictive or obstructive pattern).

**Table 1:** Anthropometric and metabolic characteristics of participants according to microalbuminuria (MAU).

	Men (n = 2530)				Women (n = 3377)			
	non-MAU (n = 2222)	MAU (n = 308)	*P*	* *P*	non-MAU(n = 2930)	MAU(n = 296)	*P*	* *P*
*Age, mean ± STD*	57.1±10.9	60.8±11	<.001		57.7±10.6	61.8±10.9	<.001	
*Pulmonary function pattern, n (%) or mean ± STD*								
normal	1496 (67.33)	162 (52.6)			2480 (84.6)	228 (77.0)		
restrictive	227 (10.2)	62 (20.1)	<.001		273 (9.3)	42 (14.2)	0.004	
obstructive	499 (22.5)	84 (27.3)	0.002		177 (6.0)	26 (8.8)	0.033	
% predicted FVC, %	91.8±11.3	87±12.2	<.001	<.001	93.6±11.5	91.1±12.1	<.001	0.004
% predicted FEV_1_, %	89.8±13.4	86.6±14.5	<.001	<.001	94.1±13.2	93.3±14.1	0.365	0.282
*Blood pressure, n (%) or mean ± STD*								
normal	638 (28.9)	33 (10.8)	<.001		1160 (39.8)	48 (16.2)	<.001	
pre-hypertension	691 (31.3)	74 (24.0)			727 (25.0)	54 (18.2)		
hypertension	881 (39.9)	200 (65.2)			1025 (35.2)	194 (65.5)		
systolic blood pressure, mmHg	123.2±15.3	130.2±16.6	<.001	<.001	121.4±16.9	132.2±19.4	<.001	<.001
diastolic blood pressure, mmHg	79.4±10.4	80.6±11.7	0.084	0.019	75.4±9.3	79.1±11.9	<.001	<.001
*Blood glucose, n (%) or mean ± STD*								
normal	1229 (57.3)	107 (36.5)	<.001		1975 (70.0)	124 (44.6)	<.001	
glucose intolerance	659 (30.7)	72 (24.6)			565 (20.0)	85 (30.6)		
diabetes mellitus	256 (11.9)	114 (38.9)			282 (10.0)	69 (24.8)		
fasting blood glucose, mg/dL	101.9±21	117.2±36.3	<.001	<.001	97.9±20.1	110.4±27.8	<.001	<.001
*Triglyceride, mg/dL, median (interquartile range)*	131.0 (92.0–194.0)	145.0 (92.5–225.0)	0.049	<.001	108.0 (75.0–155.5)	126.0 (80.0–193.0)	<.001	<.001
*Obesity, n (%) or mean ± STD*								
normal weight	1368 (61.6)	168 (54.6)	0.055		1865 (63.7)	147 (50.0)	<.001	
under weight	24 (1.1)	3 (1.0)			43 (1.5)	5 (1.7)		
over weight	830 (37.6)	137 (44.5)			1022 (34.9)	144 (48.7)		
body mass index	24.3±2.7	24.8±2.9	0.001	<.001	24.2±3.1	25.3±3.7	<.001	<.001
*Smoking history, n (%)*								
non-smoker	414 (18.6)	44 (14.3)	0.134		2717 (92.7)	273 (92.2)	0.951	
former smoker	1057 (47.6)	148 (48.1)			112 (3.8)	12 (4.1)		
current smoker	751 (33.8)	116 (37.7)			101 (3.5)	11 (3.7)		
*Heavy alcohol consumption, n (%)*	425 (19.1)	47 (15.3)	0.103		68 (2.3)	4 (1.4)	0.406	
*Physical activity, n (%)*	434 (19.5)	56 (18.2)	0.574		475 (16.2)	51 (17.2)	0.651	

*P: P values for age adjusted comparison.

Abbreviation: MAU, microalbuminuria; FVC, forced vital capacity; FEV1, forced expiratory volume in one Second; STD, standard deviation.

MAU are defined at urine albumin/creatinine ratio >30mg/g in spot urine sampling.

Man Whitney U test were used for p value of triglyceride, chi-square test and two way T-test were used for other variables.

Physical activity: Regular physical activity is defined when the participant do >20 minutes and 3 times per week or more physical activity which causes sweating.

Heavy alcohol consumption: Severe alcohol drinker is defined when the participants drink 7 glasses or more alcohol per one time, and 2 times or more alcohol drinking per a week in men (same as in men, 5 glasses or more in women is defined sever alcohol drinker).

## Results

### Basic Characteristics of Participants According to MAU (Table 1)

Participants with MAU of both sexes were older than those without MAU (mean ± SD; 57.1±10.9 y vs. 60.8±11 y, *P*<0.001 in men; 57.7±10.6 y vs. 61.8±10.9 y, *P*<0.001 in women). The proportion of poor lung function patterns differed between the MAU and non-MAU groups. The proportions of restrictive and obstructive patterns were higher in the MAU group than the non-MAU group (restrictive pattern: 20.1% vs. 10.2%, *P*<0.001 in men; 14.2% vs. 9.3%, *P* = 0.004 in women; obstructive pattern: 27.3% vs. 22.5%, *P* = 0.002 in men; 8.8% vs. 6.0%, *P* = 0.033 in women). The percent predicted value of FVC in the non-MAU group was higher than that in the MAU group for both sexes (mean ± SD: 91.8%±11.3 vs. 87%±12.2, *P*<0.001 in men; 93.6%±11.5 vs. 91.1%±12.1, *P*<0.001 in women). The percent predicted value of FEV1 in the non-MAU group was also higher than that in the MAU group for men (mean ± SD: 89.8%±13.4 vs. 86.6%±14.5, *P*<0.001 in men), but not for women.

The proportion of participants with hypertension was higher in the MAU group than in the non-MAU group for both sexes (65.2% vs. 39.9%, *P*<0.001 in men; 65.5% vs. 35.2%, *P*<0.001 in women, respectively). Mean SBP level was higher in the MAU group than in the non-MAU group for both sexes (mean ± SD: 123.2±15.3 vs. 130.2±16.6, P<0.001in men, 121.4±16.9 vs. 132.2±19.4, P<0.001 in women, unit = mmHg). The proportion of participants with diabetes mellitus was greater in the MAU group than in the non-MAU group for both sexes (38.9% vs. 11.9%, *P*<0.001 in men; 24.8% vs. 10.0%, *P*<0.001 in women). Mean FBG level was higher in the MAU group than the non-MAU group for both sexes (mean ± SD: 101.9±21 vs. 117.2±36.3, *P*<0.001 in men; 97.9±20.1 vs. 110.4±27.8, *P*<0.001 in women, unit = mg/dL). Median triglyceride level was higher in the MAU group than in the non-MAU group for both sexes (median [interquartile range]: 131.0 [92.0–194.0] vs. 145.0 [92.5–225.0], *P* = 0.049 in men; 108.0 [75.0–155.5] vs. 126.0 [80.0–193.0], *P*<0.001 in women, unit = mg/dL). The proportion of overweight women was higher in the MAU group than in the non-MAU group (48.7% vs. 34.9%, *P*<0.001). No differences in the lifestyle factors of smoking history, heavy alcohol consumption, and physical activity were observed between participants with MAU and without MAU of either sex.

In age-adjusted comparisons, mean DBP level is higher in MAU group than that in non-MAU group for men (age adjusted P<0.019). But age adjusted comparison did not alter those significant associations in the others variables.

### Multivariate Regression Analyses for Urine Albumin/creatinine Ratio According to Pulmonary Function (Table 2)

An inverse relationship was observed between lung function and UACR. In an age-adjusted regression model, the regression coefficient (B) of 10% lower in predicted FVC was 11.09 in men (*P* = 0.002), which remained significant after adjustment for SBP, FBG, triglyceride level, BMI, smoking history, and heavy alcohol consumption (B = 7.52, *P* = 0.043). The relationship was not significant in women. No significant association between UACR and FEV1 was observed in either sex.

**Table 2:** Multivariate regression analyses for urine albumin/creatinine ratio by pulmonary function.

		Model I		Model II	
		B	p value	B	p value
10% lower of predict FVC	male	11.09	0.002	7.52	0.043
	female	1.86	0.094	−0.02	0.936
10% lower of predict FEV_1_	male	4.97	0.106	1.95	0.536
	female	0.79	0.425	0.16	0.876

Model I: adjusted for age.

Model II: adjusted for age, systolic blood pressure, fasting blood glucose, triglyceride, obesity (using body mass index), smoking history, physical activity, heavy alcohol drinking.

Physical activity: Regular physical activity is defined when the participant do >20 minutes and 3 times per week or more physical activity which causes sweating.

Heavy alcohol consumption: Severe alcohol drinker is defined when the participants drink 7 glasses or more alcohol per one time, and 2 times or more alcohol drinking per a week in men (same as in men, 5 glasses or more in women is defined sever alcohol drinker).

### Logistic Regression Models for MAU According to Poor Lung Function Pattern (Table 3)

When the restrictive pattern was compared to the normal pattern, the crude ORs (95%CI) for MAU were 2.60 (1.87–3.62) in men and 1.55 (1.07–2.25) in women. That relationship remained significant after adjustment for age in men (OR [95%CI], 2.30 [1.64–3.22]) but not in women. Further adjustment for hypertension, diabetes mellitus, triglyceride level, obesity, smoking history, physical activity, and heavy alcohol consumption did not attenuate that association in men (OR [95%CI], 1.90 [1.32–2.72]). The obstructive pattern was related to MAU in the crude logistic regression model (OR [95%CI]: 1.52 [1.14–2.04] in men; 1.73 [1.12–2.68] in women), but further adjustment for age attenuated that association in both sexes. Hypertension and diabetes mellitus were related to MAU in a multiple logistic regression model (OR [95%CI]: 4.01 [2.60–6.19] and 3.96 [2.89–5.41] in men; 3.18 [2.18–4.64] and 2.42 [1.71–3.42] in women). In men, heavy alcohol consumption was related to the MAU in a multiple logistic regression model (OR [95%CI]: 1.49 [1.03–2.17]).

**Table 3:** Multivariate logistic regression model and Odds ratio (95% confidence interval) of microalbuminuria according to pulmonary function test and other covariates.

		Men			Women		
		Crude	Model I	Model III	Crude	Model I	Model III
*Pulmonary* *function pattern*	normal	1−	1−	1−	1−	1−	1−
	restrictive	2.60(1.87–3.62)	2.30(1.64–3.22)	1.90(1.32–2.72)	1.55(1.07–2.25)	1.40(0.96–2.04)	1.07(0.72–1.57)
	obstructive	1.52(1.14–2.04)	1.16(0.84–1.59)	1.09(0.78–1.54)	1.73(1.12–2.68)	1.28(0.82–2.00)	1.38(0.86–2.21)
*Covariates*							
* Age*			1.03(1.01–1.04)	1.02(1.00–1.03)		1.04(1.03–1.05)	1.01(0.99–1.02)
* Blood pressure*	normal			1−			1−
	prehypertension			2.33(1.47–3.72)			1.52 (0.99–2.32)
	hypertension			4.01(2.60–6.19)			3.18(2.18–4.64)
* Blood glucose*	normal			1−			1−
	glucose intolerance			0.98(0.70–1.36)			1.85(1.36–2.53)
	diabetes mellitus			3.96(2.89–5.41)			2.42(1.71–3.42)
* Lipid profiles*	triglyceride <150 mg/dl			1−			1−
	triglyceride ≥150 mg/dl			1.24(0.94–1.64)			1.27(0.97–1.67)
* Obesity (Body mass index, BMI)*	normal (18.5∼25.0)			1−			1−
	under weight (<18.5)			1.12(0.31–4.03)			2.33(0.86–6.33)
	over weight (>25.0)			1.17(0.88–1.55)			1.19 (0.91–1.55)
* Smoking history*	non-smoker			1−			1−
	former smoker			1.01(0.68–1.47)			0.72(0.28–1.86)
	current smoker			1.45(0.97–2.16)			1.14(0.57–2.28)
* Physical activity*				1.04(0.66–1.65)			1.29(0.82–2.02)
* Heavy alcohol consumption*				1.49(1.03–2.17)			1.56(0.54–4.47)

Physical activity: Regular physical activity is defined when the participant do >20 minutes and 3 times per week or more physical activity which causes sweating.

Heavy alcohol consumption: Severe alcohol drinker is defined when the participants drink 7 glasses or more alcohol per one time, and 2 times or more alcohol drinking per a week in men (same as in men, 5 glasses or more in women is defined sever alcohol drinker).

## Discussion

Our current study shows that restrictive lung function pattern was associated with MAU, an early surrogate marker of kidney damage and atherosclerosis, in men. That relationship remained significant even after adjustment for age, hypertension, diabetes mellitus, triglyceride level, obesity (using body mass index), smoking history, physical activity, and heavy alcohol consumption. In a multivariate regression model, a linear relationship was revealed between the lower predicted FVC and UACR, which was not attenuated after controlling for conventional atherogenic risk factors such as age, SBP, FBG, triglyceride level, BMI, smoking history, and heavy alcohol consumption. To the best of our knowledge, this study is the first investigation in Asia to examine the relationship between poor lung function and MAU.

MAU and increased UACR are regarded as early predictors of kidney damage as well as atherosclerosis [20]. MAU and increased UACR indicate that blood albumin has leaked from blood vessels of the kidneys into the urine. This leakage represents vascular dysfunction, a key pathological step of vascular damage or atherosclerosis in any organ. Hence, our current finding of an inverse relationship between FVC and MAU is consistent with evidence that poor lung function increased the risk of atherosclerosis [7]. Some reports have demonstrated that the relationship between poor lung function and risk of atherosclerosis is attenuated after controlling for conventional risk factors of atherosclerosis such as hypertension, diabetes mellitus, lipid profile, and obesity [18]. Some study show that there was significant association between poor lung function and impaired fasting glucose [21]. Thus, whether poor lung function is related to MAU when both hypertension and DM are incorporated into a multivariate model remains controversial. However, the current study revealed a strong relationship between poor lung function and MAU or UACR even after controlling for age, hypertension or SBP, diabetes mellitus or FBG, triglyceride level, obesity, smoking history, physical activity, and heavy alcohol consumption.

The obstructive lung pattern manifests as COPD, and COPD has been reported to increase systemic inflammation and elevate the risk of atherogenic events [22]. The risk of cardiovascular events increases according to the severity of COPD [23]. However, in contrast to the findings of a previous report, the obstructive pattern was not related to MAU in the current study. The obstructive pattern can be re-categorized according to its severity using GOLD criteria [24], in which the mild, moderate, and severe obstructive pattern is defined as percent predicted value of FEV1>80%, >50%, and <50%, respectively. The number (and proportion) of mild, moderate, and severe cases of the obstructive pattern in the current study was 291 (49.9%), 276 (47.3%), and 16 (2.7%) in men, and 102 (50.3%), 98 (48.3%), and 3 (1.5%) in women, respectively (data not shown in any table). The number of cases of the severe obstructive pattern was insufficient to reach statistical significance in the current study. Thus, although we did not observe a relationship between the obstructive pattern and MAU, it remains unclear whether the severe obstructive pattern is related to MAU risk.

In the current study, the restrictive pattern was strongly related to MAU as an early indicator of kidney damage and atherosclerosis. Although the pathological mechanism underlying this relationship is not clear, previous reports have shown that lower FVC is linked to atherogenic diseases [13], [25]. Furthermore, some reports have found reduced FVC to be associated with a greater increase in the risk of atherogenic disease compared to severe COPD [26]. FVC is reduced in patients with massive obesity, which disrupts pulmonary compliance due to abdominal pressure [27]. Chronic medical conditions such as diabetes and hypertension are also associated with the restrictive pattern, and mortality is sharply increased when the restrictive pattern coexists with such chronic conditions [28]. Obesity, hypertension, and diabetes mellitus are linked to systemic inflammation, and thus, they are strong atherogenic risk factors. Hence, these comorbidities may partially explain the high atherogenic risk of the restrictive pattern. However, in the current study, the restrictive pattern was significantly related to MAU even after controlling for other atherogenic risk factors, including obesity, diabetes mellitus, hypertension, and the lifestyle factors of smoking, physical activity, and heavy alcohol consumption. This result suggests that the relationship between atherosclerosis and the restrictive pattern is independent of these comorbidities.

Vascular damage and endothelial dysfunction are key mechanisms of both atherosclerosis and kidney damage. Poor lung function is also related to vascular damage and endothelial dysfunction [29] through systemic inflammation [30]. However, there is a lack of evidence concerning the relationship between poor lung function and kidney damage in Asian populations, and therefore, our study provides useful evidence on this topic. Our current results suggest that poor lung function is associated with kidney damage.

Sex differences were observed in the current study. The restrictive pattern was significantly related to MAU in men but not in women. We also observed a significant linear relationship between lower FVC and UACR in men but not in women. However, we did not find any suitable explanation for these sex differences in the literature. Among participants demonstrating the restrictive pattern (n = 604; 289 men and 315 women), the proportion of cases with MAU was lower in women than in men (n [%]: 42 [13.3%] vs. 62 [21.5%], *P* = 0.008, respectively, data not shown in any table). Additionally, the percent predicted FVC of the MAU group was 87.0% in men and 91.1% in women (*P*<0.001, data not shown in any table). Therefore, the lower proportion of women with MAU and relatively better lung function in women than in men may partially explain the lack of statistical significance in women. However, to elucidate the relationship between poor lung function and MAU in women, a large sample size including participants with poor lung function is needed.

The current study has several limitations. Due to the cross-sectional study design, the direction of causality in our study is not clear. However, evidence such as the “spilling over” theory supports our current results. To summarize the “spilling over” theory, inhaled particles cause lung inflammation and oxidative stress. When compensations to reduce inflammation and oxidative stress are insufficient, inflammatory mediators spill over from the lung to systemic circulation, subsequently exacerbating pathological change in extrapulmonary organs and causing chronic disease such as ischemic heart disease, osteoporosis, anemia, and depression [31]. Because systemic inflammation is a critical mechanism underlying atherosclerosis, the hypothesis that the relationship between poor lung function and MAU is mediated by “spilling over” of inflammatory mediators from the lung to systemic circulation, including kidney vessels, is biologically plausible. Hence, our current results are consistent with evidence of a relationship between poor lung function and the risk of vascular damage. Furthermore, our study revealed for the first time that the scope of this vascular damage extends to kidney damage. Low density lipoprotein and high density lipoprotein are important lipid profiles for risk of atherosclerosis. However, we used only triglyceride level to adjust lipid profiles, because we have no data of other lipid profiles. Hence, further research using more lipid profiles is needed to elucidate the relationship between poor lung function and MAU.

### Conclusion

Our study, the first investigation in Asia to report a relationship between poor lung function and MAU, demonstrated that the restrictive pattern is related to MAU, an early indicator of kidney damage and atherosclerosis, in men. Thus, our current study suggests that poor lung function, particularly the restrictive pattern, is related to kidney damage as well as atherosclerosis. Furthermore, the association remained significant after controlling for conventional atherogenic risk factors such as age, hypertension, diabetes mellitus, lipid profile, obesity, smoking history, and heavy alcohol consumption.
